# Identifying genomic variant associated with long QT syndrome type 2 in an ecuadorian mestizo individual: a case report

**DOI:** 10.3389/fgene.2024.1395012

**Published:** 2024-06-18

**Authors:** Rafael Tamayo-Trujillo, Rita Ibarra-Castillo, José Luis Laso-Bayas, Patricia Guevara-Ramirez, Santiago Cadena-Ullauri, Elius Paz-Cruz, Viviana A. Ruiz-Pozo, Nieves Doménech, Adriana Alexandra Ibarra-Rodríguez, Ana Karina Zambrano

**Affiliations:** ^1^ Centro de Investigación Genética y Genómica, Facultad de Ciencias de la Salud Eugenio Espejo, Universidad UTE, Quito, Ecuador; ^2^ Clinical Cardiac Electrophysiologist, Quito, Ecuador; ^3^ Instituto de Investigación Biomédica de A Coruña (INIBIC)-CIBERCV, Complexo Hospitalario Universitario de A Coruña (CHUAC), Sergas. Universidad da Coruña (UDC), Coruña, Spain; ^4^ Grupo de investigación identificación Genética-IdentiGEN, Facultad de Ciencias Exactas y Naturales (FCEN), Universidad de Antioquia, Medellín, Colombia

**Keywords:** implantable defibrillator, long QT syndrome, NGS, *kcnh2*, case report

## Abstract

**Introduction:**

Long QT syndrome (LQTS) is an autosomal dominant inherited cardiac condition characterized by a QT interval prolongation and risk of sudden death. There are 17 subtypes of this syndrome associated with genetic variants in 11 genes. The second most common is type 2, caused by a mutation in the *KCNH2* gene, which is part of the potassium channel and influences the final repolarization of the ventricular action potential. This case report presents an Ecuadorian teen with congenital Long QT Syndrome type 2 (OMIM ID: 613688), from a family without cardiac diseases or sudden cardiac death backgrounds.

**Case presentation:**

A 14-year-old girl with syncope, normal echocardiogram, and an irregular electrocardiogram was diagnosed with LQTS. Moreover, by performing Next-Generation Sequencing, a pathogenic variant in the *KCNH2* gene p.(Ala614Val) (ClinVar ID: VCV000029777.14) associated with LQTS type 2, and two variants of uncertain significance in the *AKAP9* p.(Arg1654GlyfsTer23) (rs779447911), and *TTN* p. (Arg34653Cys) (ClinVar ID: VCV001475968.4) genes were identified. Furthermore, ancestry analysis showed a mainly Native American proportion.

**Conclusion:**

Based on the genomic results, the patient was identified to have a high-risk profile, and an implantable cardioverter defibrillator was selected as the best treatment option, highlighting the importance of including both the clinical and genomics aspects for an integral diagnosis.

## 1 Introduction

Long QT syndrome (LQTS) is a predominantly autosomal dominant inherited cardiac channelopathy characterized by QT interval prolongation and T-wave abnormalities (Schwartz et al., 2012; Wallace et al., 2019). LQTS occurs in young individuals without structural heart abnormalities and is associated with syncope, ventricular tachycardia (VT), ventricular fibrillation (VF), and sudden cardiac death (SCD) (Schwartz et al., 2012; Fonseca and Joaquim, 2018). Additionally, ethnic differences in LQTS have been described. For instance, Lopez. et al. (2018) found that Hispanic patients were more likely to carry complex polygenic mutations, have longer corrected QT (QTc), and be diagnosed at a younger age compared to Caucasians (Lopez et al., 2018). Moreover, specific alleles have been described in different ethnic groups, like an increased *KCNH2* mutation frequency in Caucasians (Kong et al., 2017). Therefore, race and ancestral components could be risk factors associated with specific clinical phenotype.

LQTS has been classified into 17 subtypes according to genetic variations in 11 associated genes (Wilde et al., 2022a). Most of them affect potassium, sodium, or calcium channel function leading to cardiac channelopathies and arrhythmogenesis (Wallace et al., 2019). For instance, mutations in the *KCNH2* gene have been associated with LQTS type 2 (LQT2) (OMIM ID: 613688) (Nagaoka et al., 2008; Sakaguchi et al., 2008). The *KCNH2* gene encodes a potassium channel pore-forming alpha subunit, which forms a tetrameric ion channel composed of four copies of the KCNH2 protein and one copy of the KCNE2. Moreover, the protein is mainly expressed in tissues such as nerve cells and cardiac muscle (Safran et al., 2021).

The first-line treatment for symptomatic individuals with LQT2 is beta-blockers. However, patients with a high risk of SCD may require an implantable cardioverter defibrillator (ICD), but selecting those who will benefit from this therapy may be challenging, especially for the youngest (Cho, 2016a; Wilde et al., 2022b). We report the case of a female adolescent diagnosed with LQT2, in whom the identification of a mutation p.(Ala614Val) (ClinVar ID: VCV000029777.14) in the *KCNH2* gene, the persistence of cardiac events under beta blocker treatment, and an increased SCD risk, allowed the configuration of a high-risk profile aiding the decision to implant an ICD.

## 2 Case presentation

### 2.1 Clinical phenotype

**FIGURE 1:**
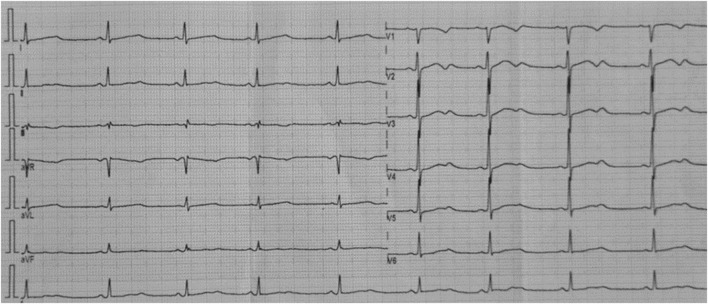
Twelve-lead rest electrocardiogram of the analyzed patient. Corrected QT interval 528 msec (Bazett formula). Flattened and notched T waves are diffusely present.

**FIGURE 2:**
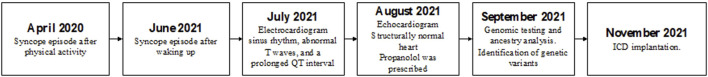
Participant’s episodes of care timeline. The disease associated episodes of care are presented in the figure.

A 14-year-old Ecuadorian female was referred to the Arrhythmia Unit for evaluation after two episodes of loss of consciousness. The first event occurred 30 min after practicing sport; while resting, she described a brief period of dizziness before losing consciousness and falling unconscious from the bed. She recovered immediately, feeling dizzy with visual dimming, pale, and diaphoretic; micturition occurred during the event. The second episode was after getting out of bed in the morning; no prodromal symptoms were reported before losing consciousness and falling; the recovery was rapid, and micturition also was present. The subject’s father shows cardiac symptoms; however, he does not have a definitive diagnosis and has refused to participate in the study. Similarly, no other family members agreed to participate in the research. Her physical exam was unremarkable. The electrocardiogram showed sinus rhythm, abnormal T waves, and a prolonged QTc interval (528 msec) (Figure 1). In the stress test, at recovery, she developed sinus tachycardia conducted with aberrancy, and when the QRS normalized, the QTc was normal (390-411 msec). The echocardiogram confirmed a structurally normal heart. According to the Schwartz score, a high probability of LQTS was estimated (5 points) (Wilde et al., 2022b). Propranolol was prescribed; however, even under treatment, the patient suffered from cardiac symptoms with increased SCD risk. Approval by the Ethics Committee of Universidad UTE, the participant informed assent and legal guardian consent were obtained. A genomic test was performed in accordance with the Declaration of Helsinki and relevant guidelines (Supplementary Figure S1). A pathogenic variant in the *KCNH2* gene p.(Ala614Val) was detected, which along with abnormal electrocardiogram confirm the diagnosis of LQT2 syndrome. The high SCD risk observed in this proband allows the ICD implantation as the best treatment option based on genomic results and lack of treatment response (persistent VT and prolonged QTc). After ICD implantation, lifestyle changes, adequate serum K^+^ levels maintenance, exercise restriction, and a reduction in auditory triggers were recommended (Cho, 2016b; Goldstein et al., 2017). Figure 2 presents a timeline with the episodes of care history.

### 2.2 Sampling and DNA extraction

The legal representative signed the informed consent, and the patient gave her informed assent to participate in the study.

A peripheral blood sample was collected in an EDTA tube. The DNA was extracted with PureLink Genomic DNA Mini Kit (Life Technologies) according to the manufacturer’s protocol (Invitrogen, 2024).

The DNA quantity and purity were measured by spectrophotometry and fluorometric methods. The quality was analyzed with an agarose gel. The sample was then diluted to 5 ng/μL according to the sequencing protocol.

### 2.3 Next-Generation Sequencing (NGS)

The genomic procedure was performed in the Genetics and Genomics Research Center of UTE University. A commercially available kit was used to identify the possible presence of variants and their association with the clinical diagnosis. We used the TruSight Cardio kit (Illumina) covering 174 genes (575 kb of cumulative target region size) with known association of 17 Inherited Cardiac Conditions (between them fifteen genes for LQTS). The samples were processed according to the manufacturer’s protocol (Illumina, 2016b). In the last step, one pool was built, denatured, and diluted to load in a V2 cartridge. The cartridge and the flow cell were inserted into the MiSeq System (Illumina) at the laboratory and run for 315 cycles.

### 2.4 Ancestry proportion analysis

For ancestral proportion analyses, PCR amplification was done with 46 ancestral informative INDEL markers in one multiplex reaction, according to Zambrano, et al. (2019) (Zambrano et al., 2019). Fragment separation and detection were executed on the 3500 Genetic Analyzer (Thermo Fisher Scientific). The results were collected on Data Collection v 3.3 and analyzed in Gene Mapper v. 5 (Thermo Fisher Scientific).

### 2.5 Genomic data analysis

For data analyses, Dragen Enrichment (v. 3.9.5) was used to perform a workflow starting with: 1) alignment to the reference genome (hg38), 2) identifying genetic variants from the sequencing reads, 3) annotating those variants with relevant information, and 4) filtering and prioritizing variants that are likely to cause the patient condition. Then, the data generated were analyzed with Variant Interpreter Software (Illumina) that integrates Sorting Intolerant From Tolerant (SIFT), Polymorphism Phenotyping (PolyPhen), ClinVar, 1000 Genomes Project, ExAC, EVS, COSMIC, dbSNP, and OMIM (Illumina, 2016a).

Data was filtered in Variant Interpreter Software and Excel to correlate if there were differences.

### 2.6 Diagnostic assessment

Ancestry proportion analysis showed an 8.3%, 40.3% and 51.3% of African, European, and Native American components, respectively.

Genomic data shown that quality score was a Q30 (probability of one incorrect base call in 1.000 bases) of 97.23%. Moreover, 1′680.470 passing filter reads with 979.287 aligned reads (78′436.355 aligned bases). Besides, the length of targeted reference (hg38) was 571.899 bp, and the depth coverage was 90.14% for 30X or above. The fragment length median was 74 bp, minimum 35 bp, and maximum 219 bp (SD = 29 pb).

The genomic pipeline of variant filter analysis started with 357 variants; the first variants that passed the quality control resulted in 350 variants. The subsequent filter was the consequence, leaving the variants that showed to be pathogenic, likely pathogenic, and uncertain significance (US); as a result, three variants were found (Table 1). Lastly, the patient was heterozygous for all the variants presented.

**TABLE 1:** Genetic variants detected in the patient diagnosed with congenital long QT syndrome.

Gene	Position/NCBI References sequence	Nucleotide/Protein change	Variant type	SIFT/PolyPhen	dbSNP ID	ClinVar
*KCNH2*	chr7:150951552/NM_000238.4	c.1841C>T p. (Ala614Val)	Missense variant	D/PD	rs199472944	Pathogenic
*AKAP9*	chr7:92042078/NM_005751.5	c.4960_4961del p. (Arg1654GlyfsTer23)	Frameshift Indels	NA/NA	rs779447911	Not Reported (US)
*TTN*	chr2:178532658/NM_173,648.4	c.103957C>T p. (Arg34653Cys)	Missense variant	NA/PD	rs773002407	US

D, deleterious; PD, probably damaging; NA, not available; US, uncertain significance.

The genomic test detected a missense variant c.1841C>T; p. (Ala614Val) in the *KCNH2* gene, which was a known pathogenic variant associated with LQT2. This result was thoughtfully discussed with the patient and her parents, and an ICD implantation was decided based on: the presence of syncope while resting, which is considered an ominous symptom, female gender, location of the variant, and lack of treatment response.

## 3 Discussion

The LQTS is a primary inherited cardiac arrhythmia syndrome that may cause SCD. An estimate of its prevalence is 1:2000 (Schwartz et al., 2009). The usual mode of inheritance is autosomal dominant except for the autosomal recessive Jervell–Lange–Nielsen type (Wallace et al., 2019). LQTS has been described in all ethnic groups and its genotype depends on the harbored genetic variant. Also, each ethnic group with LQTS is associated with a specific more prevalent genetic variant. For example, genetic variants in the *SCN5A* gene of individuals with LQTS are more prevalent in African American people, increasing the risk of sudden death (Killen et al., 2010), while Brugada syndrome (BrS) associated with *SCN5A* variants have been reported more frequently in Caucasians and Asians individuals (Milman et al., 2019; Chen et al., 2020; Nakano and Shimizu, 2022), although BrS phenotype is more prevalent in East and Southeast Asia (Nakano and Shimizu, 2022; Khawaja et al., 2023). Instead, genetic variants in the *KCNQ1* and *KCNH2* genes have been described to be more prevalent in people with European and Japanese ancestry (Lahrouchi et al., 2020; Winbo et al., 2020). A missense variant p. (Ala614Val) in the *KCNH2* gene (also known as hERG gene) have been described in several ethnic groups as a cause of LQT2. The clinical presentation of LQT2 harboring this variant is abnormal echocardiogram (prolonged QTc >500 msec), seizures, arrhythmic events and SCD (Moss et al., 2002; Tenenbaum et al., 2008). The subject described in the present case report harbors: a pathogenic variant p. (Ala614Val) in the *KCNH2* gene, prolonged corrected QT interval (528 msec), propranolol resistance, and high SCD risk, which allows the diagnosis of LQT2. However, we could not evaluate the genomic data of the proband’s family, therefore we are not able to determine if this pathogenic variant is inherited.

In Ecuador, few studies have reported ancestry analyses and cardiovascular diseases (Cadena-Ullauri et al., 2022; Guevara-Ramírez et al., 2023). In the present case report, the proband shows a high European proportion (40.3%) in ancestry proportion analysis. This high European proportion could be a risk factor, due to increased *KCNH2* mutation rates reported in European-descent population (Kong et al., 2017). However further studies should be performed to understand the ancestry role in LQTS development in the mestizo Ecuadorian population.

**FIGURE 3:**
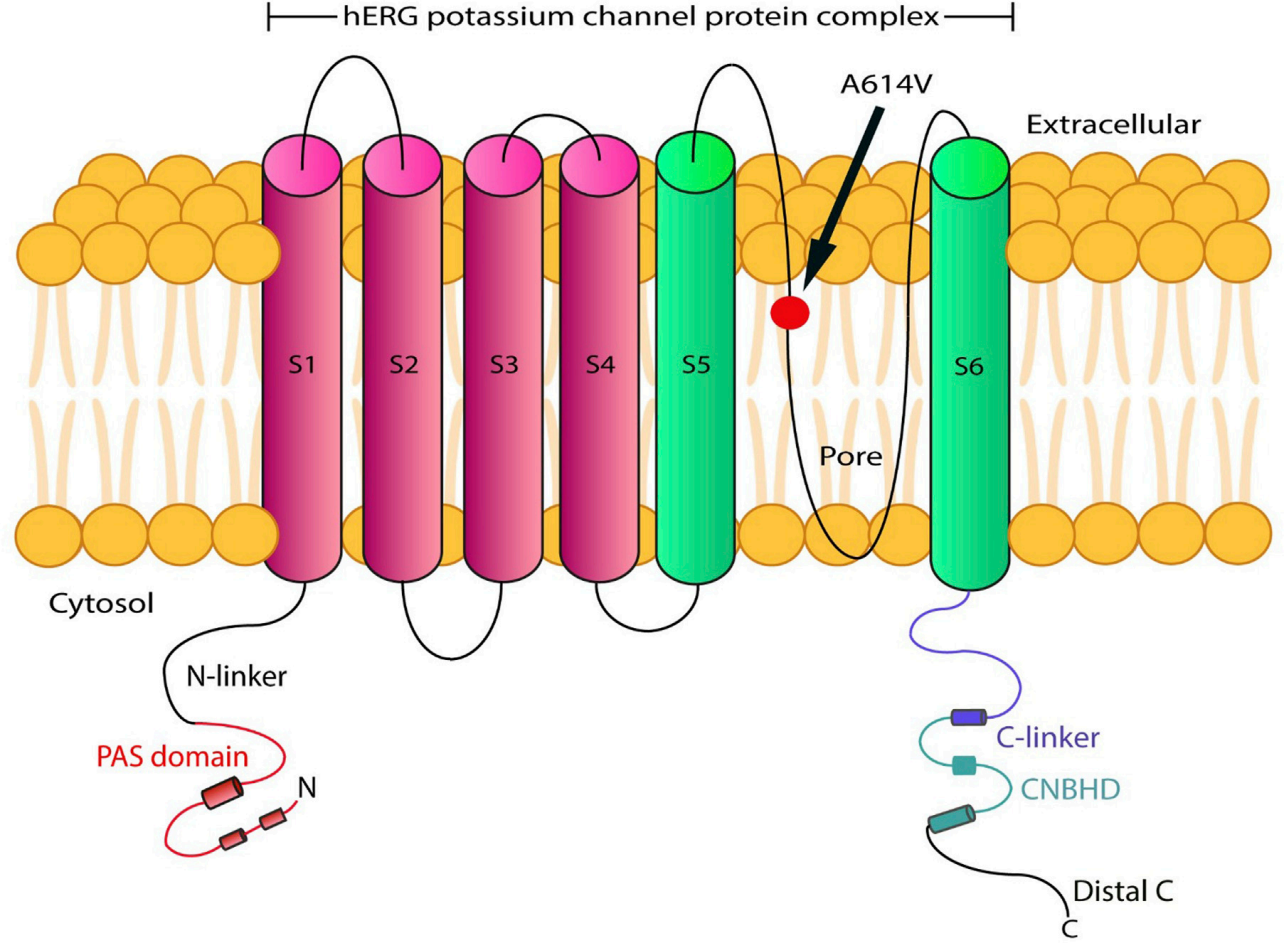
Schematic representation of the hERG protein variant (p.(Ala614Val)), located at the transmembrane pore region.

The various types of LQTS are associated with pathogenic variants in genes encoding distinct cardiac ion channels. The most common types are LQT1, LQT2, and LQT3, accounting for 85% of all genotyped LQTS cases (Wallace et al., 2019). LQT2 affects 25%-30% of LQTS individuals and is caused by heterozygous pathogenic variants in the *KCNH2* gene. This gene encodes the voltage-gated K^+^ (Kv) channel α-subunit Kv11.1. Four Kv11.1 α-subunits co-assemble into a tetrameric ion channel that conducts the rapid delayed rectifier K^+^ current (IKr) on the heart. Loss-of-function *KCNH2* mutations decrease IKr in LQT2. The impact on IKr in LQT2 depends on the variant. If the coding sequence involves amino acid residues located in the S5-loop-S6 region (552 through 657), these are considered pore variants that have been linked to an increased risk for arrhythmic events (Moss et al., 2002; Platonov et al., 2018). The pathogenic variant p. (Ala614Val) in the *KCNH2* gene shows a dominant negative effect (Anderson et al., 2014; ClinVar, 2024). Hence, in this case, the function of the potassium channel is affected by the mutant protein. The subtypes of LQTS are associated with pathogenic variants in genes involved in sodium or calcium channel proteins, as well as proteins that interact with these ionic channels (Wallace et al., 2019). Therefore, the p. (Ala614Val) variant in the pore region of the *KCNH2* gene, along with the prolonged QTc interval observed in this proband, allowed the diagnosis of LQT2 (Figures 1, 3).

Platovov et al. also showed that LQT2 female carriers with abnormal T-wave morphology had a significantly higher risk of cardiac events compared to LQT2 female carriers with normal T waves (HR = 3.31; 95% CI: 1.68–6.52; *p* = 0.001) (Platonov et al., 2018). These electrocardiographic changes were observed in our patient (Figure 1).

In addition to the *KCNH2* gene variant, the patient had a mutation in the *AKAP9* gene c.4960_4961delAG; p. (Arg1654GlyfsTer23) (rs779447911) classified as a variant of uncertain significance (VUS) but the *in silico* predictions indicate likely pathogenic effects. This gene encodes for an isoform of kinase-A anchoring protein (AKAP9), which forms a complex with the α subunit of voltage-gated potassium channel (KCNQ1) and associates with ß subunits (KCNE1), that control the slowly activating delayed rectifier potassium channels (IKs). Pathogenic variants in this gene have been associated with the progress of long QT syndrome type 1 (de Villiers et al., 2015).

The variant c.4960_4961delAG; p. (Arg1654GlyfsTer23) reported in the present study has not been previously associated with LQTS. Further studies should be performed to understand the role of this variant and its influence on the patient’s risk of fatal events. Notably, the *AKAP9* missense variant p. (Ser1570Leu) has been linked with LQTS through its involvement on IKs dysregulation. This mutation disrupts the KCNQ1-AKAP9 interaction and reduces KCNQ1 phosphorylation by cAMP induction (Chen et al., 2007). Additionally, the nonsense mediated decay (NMD) pathway could be implicated in the *AKAP9* mRNA degradation, due to earlier stop codon detected in this gene (Lindeboom et al., 2019); hence, due to the heterozygous pattern of this gene variant, the lack of AKAP9 protein could also disrupts the KCNQ1-AKAP9 interaction (Chen et al., 2007). The reported premature stop codon in the *AKAP9* gene could be implicated in the disruption of this interaction, promoting the IKs prolongation in this patient and worsening the proband’s phenotype (loss of consciousness). Therefore, *KCNH2* and *AKAP9* pathogenic variants observed in this patient, could explain the persistent cardiac events (IKr-IKs dysfunction, VT, prolonged QTc) under Propranolol therapy. Some studies have suggested that the combination of genetic variants and the additive effect of mutations in different genes can probably trigger a more aggressive phenotype in cardiac pathologies (Coll et al., 2018). Hence, all these risk factors aided the decision of ICD implantation. Recently, the link between *AKAP9* gene pathogenic variants and LQTS development has been questioned (Adler et al., 2020). However, this report could provide evidence about the role of *AKAP9* gene in potassium channelopathies.

Similarly, a missense variant in the *TTN* gene c.103957C>T p. (Arg34653Cys) (ClinVar ID: VCV001475968.4), classified as VUS, has not been previously described in LQT2. However, several nonsynonymous variants in this gene have been associated with QT interval variation and act as a risk factor for cardiac arrhythmias and SCD (Kapoor et al., 2016).

The use of genomic tools allows the identification of rare variants in a specific population. The role of these rare variants could influence in the subject clinical phenotype, due to its possible effects in cellular functional processes like signaling pathways. Moreover, the detection of rare variants could encourage the development of pharmaceutical strategies that lead to the personalized medicine approach (Momozawa and Mizukami, 2021). Therefore, the presence of the genetic variants in the *AKAP9* and *TTN* genes described in this case report, could be associated with an increased pathogenicity of the LQT2 syndrome detected in this proband. However, functional analysis of these variants must be performed to assess its effects in congenital cardiovascular diseases of Ecuadorian population.

## 4 Conclusion

Genomic and genetic tests constitute powerful tools to evaluate the predisposition and diagnosis of LQTS and other inheriting cardiac disorders associated with SCD risk and may contribute to the treatment decision-making process, especially when this process involves complex and risky procedures.

## Data Availability

The data presented in the study are deposited in NCBI Sequence Read Archive (SRA) with the BioProject accession number PRJNA917705. (https://www.ncbi.nlm.nih.gov/bioproject/PRJNA917705/). For more information, please contact the corresponding author AZ (anazambrano17@hotmail.com).
