# Supplementary figures and images for: Identifying genomic variant associated with long QT syndrome type 2 in an ecuadorian mestizo individual: a case report
Source: Front Genet. 2024 Jun 18;15:1395012. doi: 10.3389/fgene.2024.1395012 (PMC11217513; doi:10.3389/fgene.2024.1395012)

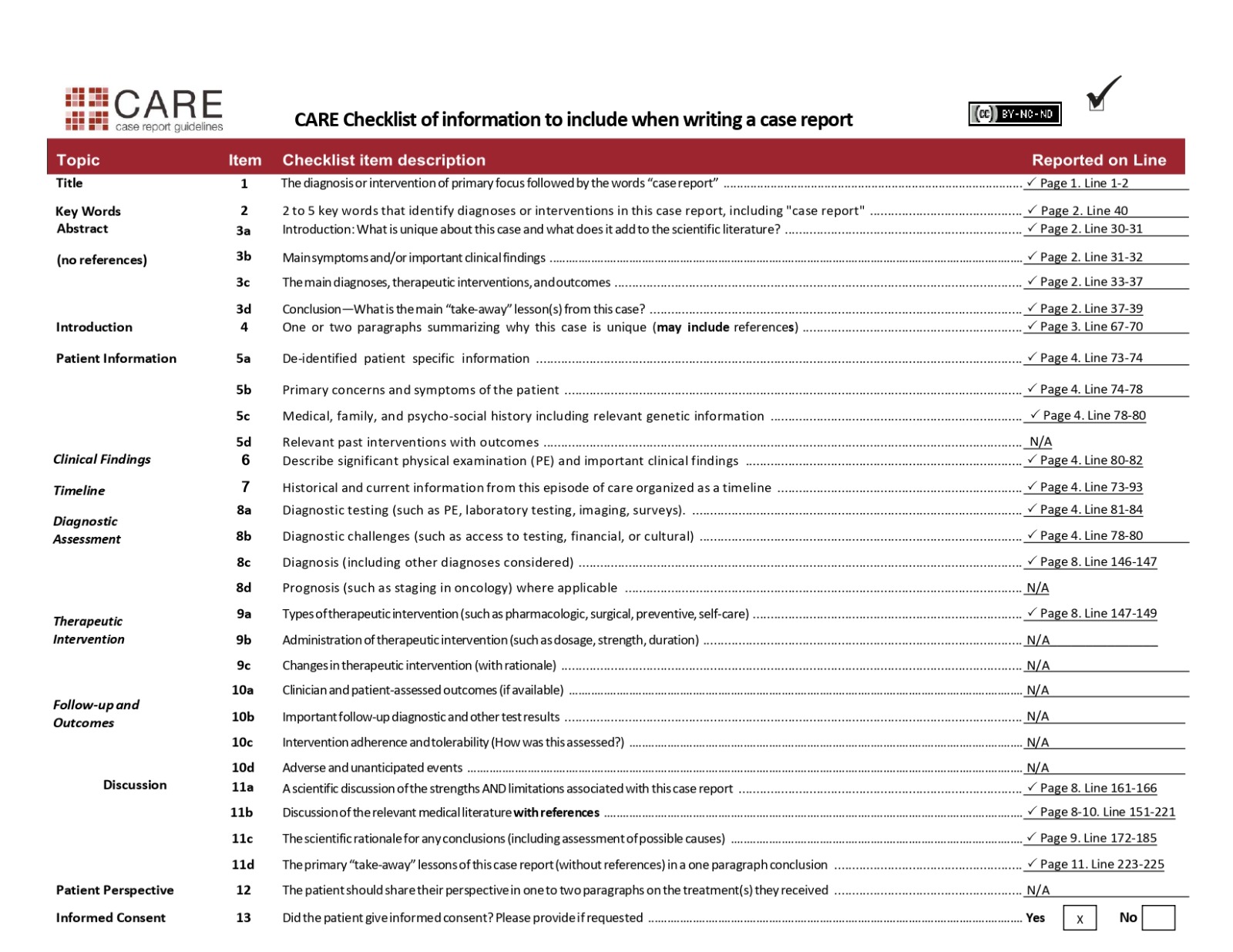

Supplement: Supplementary file 1 [file Image1.JPEG]
